# Mindful "Vitality in Practice": an intervention to improve the work engagement and energy balance among workers; the development and design of the randomised controlled trial

**DOI:** 10.1186/1471-2458-11-736

**Published:** 2011-09-27

**Authors:** Jantien van Berkel, Karin I Proper, Cécile RL Boot, Paulien M Bongers, Allard J van der Beek

**Affiliations:** 1 Department of Public and Occupational Health - EMGO Institute for Health and Care Research, VU University Medical Center, van der Boechorststraat 7, 1081 BT Amsterdam, The Netherlands; 2 Body@Work, Research Center on Physical Activity, Work and Health, TNO-VU University Medical Centre, Amsterdam, the Netherlands; 3 Department of Work and Employment, TNO Quality of Life, Hoofddorp, the Netherlands

**Keywords:** Work Engagement, Energy balance related behaviours, Physical Activity, Sedentary behaviour, Dietary behaviour, RCT, Mindfulness

## Abstract

**Background:**

Modern working life has become more mental and less physical in nature, contributing to impaired mental health and a disturbed energy balance. This may result in mental health problems and overweight. Both are significant threats to the health of workers and thus also a financial burden for society, including employers. Targeting work engagement and energy balance could prevent impaired mental health and overweight, respectively.

**Methods/Design:**

The study population consists of highly educated workers in two Dutch research institutes. The intervention was systematically developed, based on the Intervention Mapping (IM) protocol, involving workers and management in the process. The workers' needs were assessed by combining the results of interviews, focus group discussions and a questionnaire with available literature. Suitable methods and strategies were selected resulting in an intervention including: eight weeks of customized mindfulness training, followed by eight sessions of e-coaching and supporting elements, such as providing fruit and snack vegetables at the workplace, lunch walking routes, and a buddy system. The effects of the intervention will be evaluated in a RCT, with measurements at baseline, six months (T1) and 12 months (T2). In addition, cost-effectiveness and process of the intervention will also be evaluated.

**Discussion:**

At baseline the level of work engagement of the sample was "average". Of the study population, 60.1% did not engage in vigorous physical activity at all. An average working day consists of eight sedentary hours. For the Phase II RCT, there were no significant differences between the intervention and the control group at baseline, except for vigorous physical activity. The baseline characteristics of the study population were congruent with the results of the needs assessment. The IM protocol used for the systematic development of the intervention produced an appropriate intervention to test in the planned RCT.

**Trial registration number:**

Netherlands Trial Register (NTR): NTR2199

## Background

Over the last decades, technological developments have led to a change in daily working life in most sectors in the western European countries. The nature of work has become less physical, and more mentally and emotionally demanding. This is due to increasing automation, globalization and consequences such as increased pressure at work, competition, work pace, and job instability [1-3]. The change in daily working situations may contribute to adverse health effects, including mental health issues and body weight gain. The consequences of impaired mental health at work are serious, not only for the individual, but also for the organisation and society as a whole. Mental health problems are the second most frequent cause of absenteeism from work in Europe after musculoskeletal disorders [4,3]. Globally, it is one of the leading causes for disability [3].

Work can also contribute in a positive way to mental health, as it provides psychological development, time structure, social contacts, a purpose in life and it increases self-esteem and quality of life [1,3]. In general, employment is important for general mental and physical health [5,3].

Another positive aspect, related to the individual worker, is work engagement. Work engagement is a concept from positive psychology and has been defined as a "positive, fulfilling, work-related state of mind that is characterised by vigour, dedication and absorption" [6,7]. It has been shown that employees with a high level of work engagement have lower scores on burnout, anxiety and depression [8,9]. Thereby, work engagement is beneficial for organisations and society as a whole. Previous studies have shown that high work engagement was related to a lower frequency and duration of work absenteeism [10]. In addition, higher levels of work engagement were associated with higher financial return [11] and better job performance [12].

As a result of the aforementioned changes in daily working life, work has become more sedentary. This affects one side of the energy balance: energy expenditure (i.e. less physical activity behaviour). Besides a decrease of physical activity, sedentary behaviour has increasingly been found to be independently associated with overweight and obesity [13]. Together with a higher or even stable energy intake, less energy expenditure results in an energy imbalance, which leads to body weight gain and subsequently, the development of overweight [14]. Overweight and obesity have serious health and economic consequences associated with an increased risk of numerous severe diseases [15] and increased health care costs [16]. In addition, it has been shown that overweight was associated with lower levels of productivity [17,18], higher rates of sick leave [19,20], and an increased risk of the need for a disability pension [21].

In summary, it is evident that promoting mental health and a healthy body weight is potentially beneficial for individual workers, as well as for their employers. To date, and to the best of our knowledge, interventions aimed at both work engagement and energy balance-related behaviours (EBRB) do not yet exist. Given the potential effect on two important threats to the health of the workforce, the aim of the Mindful Vitality in Practice (VIP) study is to develop and evaluate an intervention targeting this combination of work engagement and EBRB to improve the health and performance of the office worker. This paper describes Phase I of the study, the systematic development of the intervention as well as the study design and baseline characteristics of the study population for the Phase II randomised controlled trial (RCT).

## Methods/Design

### Methods for Phase I

#### The Systematic Development of the Intervention

The intervention was developed in a systematic way, based on the Intervention Mapping (IM) approach [22]. IM describes a stepwise process for theory- and evidence-based development of a health promotion intervention. The following six steps were followed: 1) needs assessment, 2) definition of program objectives, 3) selection of adequate methods and strategies to realise behavioural change, 4) program development, 5) development of a plan for implementation, and 6) evaluation.

#### Step 1: Needs Assessment

For this first step, the needs of the target population were assessed by:

1) exploring the work engagement and selecting EBRB of the target population: highly educated workers of two public research institutes

2) exploring the key resources and determinants of work engagement and EBRB of the target population.

1) A literature search was performed, face-to-face interviews were held, and a brief questionnaire was distributed among workers to gain insight into the work engagement and EBRB of the target population.

The mean score for work engagement for Dutch workers is 3.82, measured in a representative sample of Dutch workers using the Utrecht Work Engagement Scale (UWES), on a scale from zero to six, where zero is the most negative and six the most positive [7]. No specific data on work engagement are known for the governmental sector, scientific professions or higher educational levels.

Dutch workers, on average, spend about seven hours total per day sitting--one third of this at work [23]. Sedentary behaviour at work can not be compensated by increased physical activity in leisure time. Furthermore, workers in the governmental sector (to which the target group belongs) are amongst the top five sectors in which workers spent significantly more time sitting at work and in total during the day than the average Dutch worker [23].

Face-to-face interviews were held with six key figures in one study research institute to gain insight into the characteristics of work engagement and EBRB of the target population. Managers, Human Resource Management officers, and staff assistants characterized the target population as highly motivated and involved workers, who spent a lot of time on cognitive activities. The key figures stated that mental well-being was more of a priority for this population than EBRB, and they believed there were more mental problems than physical ones in this group.

A brief questionnaire was sent to 100 workers from various departments in order to obtain a heterogeneous sample and to learn about the status of work engagement and relevant sub-behaviours of EBRB among the target population. Of the 100 employees approached, 78 completed and returned the brief questionnaire, which included questions about work engagement as measured by the UWES [7]. Furthermore, the questionnaire contained questions about domains and intensity of physical activity, sedentary behaviour and dietary behaviour in order to select sub-behaviours to target specifically in the intervention.

This questionnaire indicated that the work engagement score of the respondents fell in the category "average engaged". With regard to EBRB, vigorous physical activity in leisure time and sedentary behaviour at work were selected for the energy expenditure related-behaviours as it were a potential problematic but changeable behaviour. For energy intake behaviours, fruit and vegetable intake was selected as representative on the same grounds as the expenditure related behaviours.

2) Based on the outcomes of the brief questionnaire, semi-structured focus group discussions were organized around the two themes: work engagement and EBRB. The aim of the focus group discussions was to identify key resources of work engagement and key determinants of the selected EBRB. In total, six focus groups were carried out with a total of 39 workers.

The focus group discussions were recorded and transcribed and field notes were written. The data from these interviews were analysed and coded. These codes were matched to resources of work engagement and determinants of EBRB as identified in the literature. Furthermore, resources and determinants were selected for changeability determining their appropriateness as intervention targets.

##### Key resources

Conclusions from the focus group discussions combined with the literature identified the following as the most important resources of work engagement for the research population: self-efficacy, organisational-based self-esteem, optimism, and social support.

Self-efficacy, organizational-based self-esteem and optimism are personal resources. These personal resources originate from the Job Demands-Resources (JD-R) Model [24]. This model suggests that working characteristics can be classified into two broad categories: job demands and job resources. Personal resources mediate the relationship between job resources and work engagement [25].

Self-efficacy is the individual's perception of their ability to meet demands in a broad array of contexts. Organizational-based self-esteem is the degree to which workers believe that they can satisfy their needs by participating in roles within the context of the organization. Optimism is the tendency to believe that one will generally experience good outcomes in life, which increases the propensity to take action and manage threats. Social support is a job resource [24,25]. Of all resources, personal resources are the most changeable. Of job resources, social support is most changeable and therefore these are most suitable to target in an intervention.

##### Key determinants

Conclusions from the focus group discussions identified the most important determinants of vigorous physical activity in leisure time and for sedentary behaviour at work (including during lunch time) to be: perceived behavioural control, perceived barriers (especially lack of time), and social support. The intention to engage in physical activity and the awareness of sedentary behaviour were additional determinants for physical activity and sedentary behaviour, respectively. For fruit and vegetable intake, the most important determinants were: habit, perceived behavioural control, availability, cost, and intention.

Most key determinants were related to the individual. Perceived behavioural control consists of two elements: self-efficacy and controllability: the beliefs of the ability to carry out a certain behaviour and control over the behaviour. Perceived behavioural control originates from the theory of planned behaviour [26,27], as does intention. Intention is considered a key indicator of behaviour, but the intention-behaviour relationship is complex and influenced by other factors. Perceived barriers originate from the Health Belief Model [28], which reflects the perception of obstacles to perform a specific (desirable) behaviour. A lack of time is a quite commonly perceived barrier, which impedes individuals to engage in physical activity in developed countries [29]. Habit is a recurrent, often unconscious, behavioural pattern. Awareness [30] is the state or ability to be conscious of behaviour. Costs, availability and social support are environmental factors.

#### Step 2: Definition of Program Objectives

Program objectives were defined based on the results of the needs assessment (Step 1).

The formulated program objectives were: increasing work engagement, increasing vigorous physical activity in leisure time, reducing sedentary behaviour at work (including lunch time), and increasing fruit and vegetable intake.

#### Step 3: Methods and Strategies

##### Methods

In Step 3, theory-informed methods and practical strategies were chosen to improve work engagement and the selected EBRB behaviours of the target group based on the key resources and key determinants identified in the needs assessment (Step 1). A method is a theory-based technique to influence resources and determinants, whereas a strategy organises and operationalises the intervention methods.

Self-regulation skills are an important method to increase work engagement and physical activity at leisure, as well as to reduce sedentary behaviour at work. Self-regulation refers to the same construct as self-management: "an active, iterative process, of goal setting, choosing of strategies, self-observation, making judgments based on observation (as opposed to ones on habit, fear or attraction), reacting appropriately in the light of one's goal and revising one's strategy accordingly" [22](p. 127). Tables 1, 2, 3 and 4 present the methods and strategies for the program objectives. Because the key resources and determinants are generally individual, the method(s) are mainly directed at the individual including goal setting and positive reinforcement. To address environmental determinants (costs, availability, environment facilities) of fruit and vegetable intake, adaptations are made in the facilities at the workplace, such as free fruit and vegetables, and suggested lunch time walking routes. In addition, social support was mobilized as part of the methodology to improve work engagement and EBRB.

**Table 1:** Methods and strategies selected for increasing work engagement

Key resources	Theoretical method	Practical strategy	Tools and Materials
**Self efficacy**	Self regulation Goal setting Reinforcement	In-company mindfulness training Facilitate formulation of goals and reflection Providing positive feedback	Exercises aimed at enlarging sense of control over behaviour and beliefs of efficacy. E-coaching: Employees formulate goals and reflect in logbook. Coach reads and stimulates reflection In-company mindfulness training: trainer provides positive feedback E-coaching: coach provides positive feedback
**Organisational based self-esteem**	Self regulation Goal setting Reinforcement	In-company mindfulness training Facilitate formulation of goals and reflection Providing positive feedback	Exercises aimed at self esteem at work. E-coaching: Employees formulate goals and reflect in logbook. Coach reads and stimulates reflection In-company mindfulness training: trainer provides positive feedback E-coaching: coach provides positive feedback
**Optimism**	Self regulation	In-company mindfulness training	Exercises aimed at optimism
**Social support**	Mobilising social support	Form groups or pairs	Group and duo discussions in in-company mindfulness sessions to discuss exercises. Finding a buddy is stimulated during the sessions.

**Table 2:** Methods and strategies selected for increasing physical activity in leisure time of vigorous intensity

Key determinants	Theoretical method	Practical strategy	Tools and Materials
**Perceived behavioural control**	Self regulation Goal setting Reinforcement	In-company mindfulness training Facilitate formulation of goals and reflection Provide positive feedback	Exercises aimed at enlarging sense of control over behaviour and beliefs of efficacy. E-coaching: Employees formulate goals and reflect in logbook. Coach reads and stimulates reflection In-company mindfulness training: trainer provides positive feedback. E-coaching: coach provides positive feedback
**Perceived barriers**	Self regulation Goal setting	In-company mindfulness training Facilitate formulation of goals and reflection	Exercises aimed at reperceiving barriers and enacting on intentions. E-coaching: Employees formulate goals and reflect in logbook. Coach reads and stimulates reflection
**Social support**	Mobilising social support	Form pairs or groups	In-company mindfulness training in groups and oral instruction to form pairs to do discuss homework exercises
**Intention**	Self regulation	In-company mindfulness training	Exercises aimed at enacting on intentions

**Table 3:** Methods and strategies selected for reducing sedentary behaviour at work

**Key determinants**	**Theoretical method**	**Practical strategy**	**Tools and Materials**
			
**Perceived behavioural control**	Self regulation Goal setting Reinforcement	In-company mindfulness training Facilitate formulation of goals and reflection Provide positive feedback	Exercises aimed at enlarging sense of control over behaviour and beliefs of efficacy. E-coaching: Employees formulate goals and reflect in logbook. Coach reads and stimulates reflection In-company mindfulness training: trainer provides positive feedback E-coaching: coach provides positive feedback
**Perceived barriers**	Self regulation Goal setting Reinforcement	In-company mindfulness training Facilitate formulation of goals and reflection Provide positive feedback	Exercises aimed at reperceiving barriers and enacting on intentions. E-coaching: Employees formulate goals and reflect in log-book. Coach reads and stimulates reflection by providing feedback. In-company mindfulness training: trainer provides positive feedback. E-coaching: coach provides positive feedback
**Awareness**	Self regulation	In-company mindfulness training	Exercises aimed at body awareness and behavioural patterns
**Social support**	Mobilising social support	Form pairs or groups	In-company mindfulness training in groups and oral instruction to form pairs to do discuss homework exercises
**Physical environment**	Environmental change	Facilitation of reducing sedentary behaviour during lunchtime	Providing routes for lunch walking

**Table 4:** Methods and strategies selected for increasing fruit and vegetable intake

Key determinants	Theoretical method	Practical strategy	Tools and Materials
**Habit**	Self regulation Goal setting Reinforcement	In-company mindfulness training Facilitate formulation of goals and reflection Providing positive feedback	Exercises aimed at preventing counter intentional habits from obstructing enactment of intentions E-coaching: Employees formulate goals and reflect in personal e-diary on website. Coach reads and stimulates reflection E-coaching: coach provides positive feedback with regard to set goals
**Perceived behavioural control**	Self regulation Goal setting Reinforcement	In-company mindfulness training Facilitate formulation of goals and reflection Provide positive feedback	Exercises aimed at enlarging sense of control over behaviour and beliefs of efficacy. E-coaching: Employees formulate goals and reflect in logbook. Coach reads and stimulates reflection E-coaching: coach provides positive feedback
**Availability**	Environmental change	Facilitation of healthy behaviour	Offering fruit and vegetables
**Costs**	Environmental change	Facilitation of healthy behaviour	Offering fruit and vegetables
**Intention**	Self regulation	In-company mindfulness training	Exercises aimed enacting on intentions

##### Strategies

The program's self-regulation strategy was mindfulness training. Mindfulness can be described as "an open, undivided observation of what is occurring both internally and externally" [31] (p. 823). Hypothetically, mindfulness training best fits the study's target group, as it is likely to attract individuals who have a high need for cognition because they pay attention to relevant arguments [32].

Mindfulness training has been shown to be effective in improving aspects of both mental and physical health [33]. Although evidence of mindfulness training strategies to improve work engagement is lacking, evidence does exist that it can improve subjective well-being; this is relevant because work engagement can be seen as subjective well-being at work [34]. Proven effective activities to improve subjective well-being can be divided into three categories: behavioural, cognitive and motivational. A cognitive activity that can improve subjective well-being is mindfulness [34]. In the Mindful VIP study several activities were integrated in the program (see Step 4 for details). Furthermore, the effectiveness of mindfulness training to increase physical activity and to stimulate body weight reduction has been demonstrated in small scale interventions [32,35].

#### Step 4 Program Development

##### Mindfulness training

Participatory focus groups were held to develop the mindfulness training program. The groups discussed the duration of the proposed sessions and 90 minutes was considered feasible. The training will last eight weeks, as is usual for mindfulness training [36]. Each of the eight sessions will take 90 minutes, which is shorter than the usual duration (2.5 hours), but in-company (i.e., in the workplace) mindfulness training of shorter duration has also proven to be effective [37]. The mindfulness training will be given by certified trainers.

##### Cognitive components

The main cognitive component of the training is "enjoying the here and now" [31,38,34]. Specific exercises aimed at mindfulness at work will be part of the program, for instance, short breathing and meditation exercising while starting up a computer or enjoying a cup of coffee. Other cognitive activities integrated in the program are: counting your blessings [39,34] by using a log book and discussing this during the training, stimulating optimism [34] by raising awareness of positive thoughts (using a log book), and breaking negative and pessimistic thoughts and replacing them with neutral or positive thoughts.

##### Behavioural components

Mindful VIP training behavioural components will be treasuring social relations [38,34] by getting in contact with colleagues in the training, performing acts of kindness for oneself (since being in a mild and friendly state is the core of mindfulness) [31], and performing acts of kindness to others [40,34]. The program will include homework exercises, such as (sincerely) complimenting a colleague each day and noticing what this does for oneself and for others.

##### Motivational components

Goal setting and increasing resilience are the motivational components of the intervention [38,34]. Before coming to the first session of the training, participants will be asked to fill in a form to reflect on their work and lifestyle. It is expected that increasing mindfulness will increase resilience. For instance, in stressful work situations, mindful workers do not tend to get swept away by (negative) emotions and thoughts, but instead, become neutral or stronger when confronted by difficult situations since their self-efficacy is increased.

##### EBRB-targeted components

In the Mindfulness VIP training, several exercises aimed at (determinants of) EBRB will be integrated in the program as well, such as: "walking meditation", "mindful eating" and "shopping for groceries mindfully".

##### E-coaching

Mindfulness trainers will perform e-coaching to continue implementing the mindfulness principles learnt in the training. In the seventh session of the training, participants will be invited to formulate goals and think of ways to achieve them in the format of a Personal Energy Plan (PEP) as homework. Participants will be asked to return the PEP by email to the trainer. This PEP functions as the basis for the coaching; participants will monitor their personal goals and reflect on their progress in the PEP journal. To maximize the effects of the goal setting, the goals should correspond to personal values and needs [41]. To obtain a maximum of effect, the goal setting in the PEP plan will be preceded by a personal value exercise in the training. Attaining goals that match personal values not only contributes to attaining the goals, but also to subjective well-being [42]. Therefore, this contributes also to work engagement as this is a positive affective-cognitive state just like well-being, only work-related [34].

##### Supporting elements

There will be three supporting elements in this intervention: providing fruit at work, providing routes for lunch walking, and stimulation (by the mindfulness trainer) to find a buddy for several activities. This buddy can be a facilitator to attend to mindfulness sessions, help with the homework exercises for the mindfulness training, accompany the subject on lunch walking, join in exercising, share commuting to work by bike, or to talk about work and reflect on the learning process. During the mindfulness sessions, homework exercises will be discussed with a buddy.

#### Step 5 Development of a Plan for Implementation

Two additional participatory focus groups were convened to design a plan for implementation. Participants included the intended implementers as well as the recipients of the intervention. Both groups discussed the possibilities for success and potential challenges of the implementation of the intervention. The outcomes from these focus group sessions were embedded in the instruction of the trainers. It was decided to use a down-to-earth approach with participants in the intervention and to use clear language with straightforward formulated instructions and explanations. Such an approach would avoid the risk of deterring intended participants from the intervention.

#### Step 6: Evaluation

The evaluation of the effectiveness will be presented in the next section.

### Phase II: Evaluation of the Intervention

#### Study Design

The effectiveness of the Mindfulness VIP program will be measured using a RCT. Participants who have given informed consent were measured at baseline (T0), and will be measured at 6 months (T1) and 12 months (T2). The study design and procedures have been approved by the Medical Ethics Committee of the VU University Medical Center.

#### Population

The study population included employees of two Dutch research institutes in the governmental sector. Most workers are highly educated (most have completed higher vocational education or university), who perform sedentary work. All employees at both institutes were invited to participate in this study. Exclusion criteria were being on sick leave for more than four weeks or being pregnant at the time of recruitment.

#### Randomisation and Blinding

Randomisation was performed at individual level, directly after the baseline measurement (T0), using a computer-generated randomisation sequence in SPSS by an independent researcher. As a result of the nature of the intervention, blinding of the participants and the trainers was not possible.

#### Recruitment

The usual and available communication channels of the participating organisations were used to recruit participants. These channels included intranet, internet, a personnel magazine, posters on bulletin boards and digital newsletters. Efforts were made to create support from supervisors and directors. Workers were also invited by email. In one of the participating organisations, an additional mailing (hard copy) was distributed.

#### Procedure

Consenting participants were randomised to the intervention or control group after the baseline measurement. The control group as well as the intervention will be asked to complete the two follow-up measurements after 6 (T1) and after 12 months (T2).

### Primary Outcome Measurements

The primary endpoint of this study will be work engagement as measured by the Utrecht Work Engagement Scale (UWES). The UWES consists of three aspects: vigor (6 items), dedication (5 items), and absorption (6 items) [7]. The three aspects combined result in an overall work engagement score. Varying from 0 to 6; a higher UWES score indicates a higher work engagement.

### Secondary Outcome Measurements

#### Energy Balance-Related Behaviours

Physical activity was assessed by the Short Questionnaire to Assess Health Enhancing Physical Activity (SQUASH) [43]. It measures duration, frequency and intensity of work transportation, household activities, leisure activities and work activities. In accordance with the second program objective, the weekly level of physical activity of vigorous intensity in leisure time was assessed by an item documenting how often these activities were performed for at least 20 minutes.

Questions related to sedentary behaviour at work include the time spent (in minutes per day) on: 1) computer use, 2) reading, 3) meetings, 4) calling and 5) other activities. Additionally, physical activity and sedentary behaviour were measured objectively using a single waist-worn Actigraph Computer Science and Applications accelerometer. This accelerometer collected data on activity, step count and three axes (with an epoch period of 60 seconds over seven consecutive days) at baseline and at follow-up measurements among a randomly chosen subsample of 100 participants. The same 100 participants were asked to wear the same accelerometer at the follow-up measurements. To interpret the data obtained by the accelerometer adequately, participants were asked to keep a logbook of their physical activities and sedentary behaviour during the seven days of wearing the meter.

Dietary behaviour was assessed by focussing on fruit and vegetable intake using the Short Fruit and Vegetable Questionnaire [44]. The questionnaire consists of 10 questions: six for fruit consumption and four for vegetable consumption.

#### Mental and Physical Health-related Outcome Measures

Mental health, vitality and general health perceptions were all measured by the corresponding items within the RAND-36 [45]. For the Mental Health Inventory from the RAND-36, participants were asked to indicate on a six-point scale for five items how often they felt anxious, depressed, calm, sad and happy during the past four weeks. For vitality, participants were asked to indicate on a six-point scale how often they felt full of life, worn out, tired and full of energy over the past four weeks. For general health perceptions, participants were asked to indicate on five items how they perceive their health (on a five-point scale from excellent to bad) and to indicate on four propositions to what extent they agree (using a five-point scale).

##### Mindfulness

The level of mindfulness was measured using the Mindful Attention Awareness Scale (MAAS) [31]. This 15-item scale measures the frequency of everyday mindfulness experiences on a six-point scale.

##### BMI and waist circumference

All anthropometric measurements were performed by trained research assistants. Overweight and obesity are commonly measured by the body mass index (BMI). BMI is calculated by dividing body weight in kilograms by the square of the body height in meters. Height was measured to the nearest 0.1 cm without shoes. Weight was measured to the nearest 0.5 kg in participants wearing indoor clothing and no shoes, after emptying their pockets. Waist circumference was measured as midway between the lower rib margin and the iliac crest to the nearest 0.1 cm [46]. Participants were measured standing up, breathing out gently, in indoor clothing, after emptying their pockets. All measurements are repeated to increase reliability.

##### Need for recovery

The 11-item 'need for recovery scale' from the Dutch version of the Questionnaire on the Experience and Evaluation of Work (Dutch abbreviation VBBA) were used [47].

##### Measures of determinants of behaviour

Based on models of behaviour, questions were asked on perceived behavioural control, intention and perceived barriers (lack of time) for physical activity and dietary behaviours.

##### Prognostic factor

The need for cognition (prognostic factor) was assessed using the short version of the Need for Cognition Scale [48]. This scale consists of 18 items on which subjects have to indicate on a five-point scale to what extent the item is characteristic of them. Items assess engagement in and enjoyment of thinking, learning and intellectual cognitive activities.

#### Economic Measures

##### Workplace productivity loss

Sickness absence data (absenteeism) will be collected from company records after the last follow-up measurement. Presenteeism (reduced productivity while at work) was measured using the WHO Work Performance Questionnaire (WHO-HPQ)[49,50] and the PROductivity and DISease Questionnaire (PRODISQ) [51,52]. Participants were asked to complete these questionnaires at 3, 6, 9, and will be asked again at 12 months.

##### Work-related outcome measures

In addition to specific cost-measures, some work-related outcome measures will be evaluated, such as work satisfaction and organisational commitment.

##### Cost measures

Intervention costs include the costs for group meetings (i.e. costs for the trainer, housing, and materials), e-mail contacts with trainers, website hosting and maintenance, offering fruit and snack vegetables, and printed materials. Frequency and duration of group meetings and e-mail contacts were recorded by the trainers. Trainer and coach costs will be valued based on their salary. Other costs will be valued using invoices of contractors.

##### Referral costs

Trainers may refer participants to existing occupational health activities of the organisation, such as a consult with an occupational physician or with one of the in-company fitness professionals. The use of these options were reported by the participants at 6 and will be reported at 12 months, and will be valued using the contractors' invoices.

##### Health care costs

These include a general practitioner, allied health care, medical specialist, complementary medicine, hospitalisation, or medications. Data on resource use were and will be collected on a quarterly basis using retrospective questionnaires. Dutch standard costs [53] will be used to value health care utilization. If these are not available, prices according to each professional organization will be used. Medication use will be valued using unit prices provided by the Dutch Society of Pharmacy [54].

##### Productivity-related costs

Workplace productivity losses (i.e. absenteeism and presenteeism) will be valued using salaries of the participants.

##### Participant costs

Since the intervention stimulates participants to engage in regular physical activity, self-reported costs related to sports activities (membership fees and sports equipment costs) will be collected on a quarterly basis.

### Data Analysis

#### Baseline Characteristics

The baseline characteristics were analysed using descriptive statistics. All characteristics were tested for differences between the intervention and control group (with either independent sample t-test or chi-square test in case of normal distribution, and with the non-parametric alternatives in case of non-normal distribution), to determine randomization success.

#### Effect Evaluation

The effectiveness of the intervention will be analysed using a regression analysis (analysis of covariance) with the outcome measure at follow-up (T1: 6 months and T2: 12 months) as the dependent variable and adjusting for the baseline levels of the outcome measure. In addition, a Generalised Estimated Equations (GEE) analysis will be performed for the long-term effects (baseline to 12 months). All statistical analyses will be performed according to the intention-to-treat principle. For all analyses a two-tailed significance level of < 0.05 will be considered statistically significant. All analyses will be performed with SPSS 16.0 (SPSS Inc. Chicago, Illinois, USA).

#### Process Evaluation

A process evaluation will be carried out to gain insight into the factors influencing the effectiveness of the intervention program of Mindful VIP, such as the organisational context, receipt and implementation of the program.

#### Economic Evaluation

The economic evaluation aims to determine the cost-effectiveness of the intervention compared to the usual care from the societal and employer's perspective. Also, the cost-benefit analysis will be performed from the employer's perspective. The time horizon will be one year, similar to the trial. Analyses will be performed according to the intention-to-treat principle. In the main analysis, missing data will be imputed using fully conditional specification [55]. Sensitivity analyses will be done to assess the robustness of the results.

First, the total societal and employer's costs will be estimated, and compared between the intervention and control group. The 95% confidence intervals will be estimated using approximate bootstrap confidence intervals [56]. Societal costs include all cost measures described in the method section. From the employer's perspective, only costs relevant to the company are included (i.e. intervention, referral, and productivity-related costs). For the cost-effectiveness analysis (CEA), incremental cost-effectiveness ratios will be calculated by dividing the difference in costs between both groups by the difference in effects on the primary outcome measures (societal perspective) and work-related outcomes measures (employer's perspective). Bootstrapped cost-effect pairs will be graphically presented on cost-effectiveness planes [57]. Cost acceptability curves will be generated, showing the probability for cost-effectiveness of the intervention at various ceiling ratios. Also, a cost-benefit analysis (CBA) will be performed in which the incremental intervention and referral costs will be compared to the incremental productivity-related costs.

### Sample Size Calculation

The sample size was based on finding an effect on work engagement, measured using the UWES [7]. The mean score for work engagement was 3.82 (sd = 1.1) on a scale from 0-6 [7]. An effect of a 10% increase in mean score was expected to be relevant and feasible. With a power of 90% and a two-sided alpha of 5%, both groups needed 175 workers. Accounting for a loss to follow-up of 25% over 12 months, each group needed 234 workers at baseline, thus an initial total of 468 workers for the two groups.

After recruiting 200 participants, the preliminary baseline characteristics were examined. It appeared that within our study population the standard deviation was smaller than the value reported in literature (0.83). With this knowledge, the power was recalculated, leading to a group size of 89 participants. When taking into account a 25% loss to follow-up, both groups needed 119 workers, thus a total of 238 workers to be included at baseline.

### Baseline Characteristics

Informed consent was obtained from 260 participants, of whom 258 participated in the baseline measurement of BMI and waist circumference. The questionnaire was completed by 257 eligible participants, who were subsequently randomly assigned to either the control or intervention group. The intervention group had 129 participants, and the control group had 128 participants.

Table 5 summarizes the baseline characteristics for the intervention and control group. Demographic characteristics as presented in Table 5 show that the majority of the study population was female (67.3%). Most of the participants were married or had a significant other (77.4%). More than four out of five participants were highly educated (completed higher vocational education or university).

**Table 5:** Baseline characteristics

	Intervention group (n = 129)	Control group (n = 128)
**Demographics**		
Gender, Female *%*	63.56	71.09
Marital status: Married or significant other, %	81.39	73.43
Education: Highly educated*, *%*	76.74	85.93
Age, years, *m ± sd*	46.0 *± *9.4	45.1 *± *9.6
**Prognostic factor**		
Need for cognition, *m ± sd*	3.67 *± *0.51	3.75 *± *0.59
**Work engagement**		
Work engagement **, *m *± *sd*	4.09 *± *0.81	4.02 *± *0.91
Vigor, *m *± *sd*	4.23 *± *0.83	4.11 *± *0.91
Dedication, *m *± *sd*	4.39 *± *0.91	4.26 *± *0.96
Absorption, *m *± *sd*	3.77 *± *0.91	3.82 *± *0.91
**Energy Balance Related behaviours**		
Vigorous physical activity in leisure time, minutes per week, *m *± *sd*	86.32 *± *158.93	51.05 *± *127.45
Not engaging in vigorous activity in leisure time, *%*	50.40%	69.50%
Sedentary behaviour at work, minutes per working day, *m *± *sd*	496.74 ± 136.67	490.19 *± *113.96
Daily fruit intake in pieces, *m *± *sd*	1.51 *± *0.95	1.44 *± *0.93
Daily vegetable intake in grams, *m *± *sd*	183.55 *± *74.00	182.43 *± *83.36
**Weight status**		
BMI, kg/m^2^, *m *± *sd*	24.74 ± 3.96	24.66 ± 3.56
Underweight (≤ 18.49 kg/m^2^), *%*	0.80	0.00
Normal weight (18.50-24.99 kg/m^2^), *%*	63.40	59.40
Overweight (25.00-29.99 kg/m^2^), *%*	25.60	32.00
Obese (≥ 30.00 kg/m^2^), *%*	9.40	8.60
Waist circumference (cm), *m *± *sd*		
Men	91.44 ± 10.72	91.39 ± 8.67
Women	80.30 ± 10.46	80.41 ± 9.29

* Higher vocational education or university ** Measured by the UWES 17 (Schaufeli & Bakker, 2003)

The mean score for work engagement was 4.09 in the intervention group and 4.02 in the control group (Table 5). Furthermore, of all the participants, 60.1% did not engage in vigorous physical activity in leisure time at all; 0.4% was underweight (< 18.50 kg/m^2^), about 29% was overweight (BMI between 25 kg/m^2 ^and 30 kg/m^2^), and about 9% was obese (BMI > 30 kg/m^2^).

No differences were observed between the intervention and control group for any of the variables, except for vigorous physical activity (p = .002).

## Discussion

In this paper, the development of the Mindful VIP study, the design of its evaluation, and the baseline characteristics of the study population are described. The systematic development of the Mindful VIP program was based on the IM protocol, involving the target population and other stakeholders such as the management. This resulted in a program with the objective to increase work engagement, vigorous physical activity in leisure time, daily fruit and vegetable intake, and to decrease sedentary behaviour at work and during lunch time. Key resources of work engagement and key determinants of EBRB were explored for the target population. The chosen methods and strategies in the Mindful VIP program target these key resources and determinants. The program consists of an in-company mindfulness training, e-coaching, fruit and snack vegetables, a buddy system, and lunch walking routes.

The demographic characteristics related to the entire working population at the research institutes rather well in terms of age. The mean age of workers at the largest of the two research institutes was about 46 in 2010. The distribution of gender within the study population, however, differed from the entire working population at the two institutes in 2010. In our study population, there were more women (about 67%) than men, whereas in the actual working population, there are almost as many men (52%) as women. When interpreting the evaluation of the effects, this should be considered as it might have implications for the generalisability. In the process evaluation, the reach and recruitment of the target population will be further examined.

Baseline findings confirmed the findings and assumptions resulting from the needs assessment. Consistent with findings in the needs assessment and with findings in the Dutch population [7], the mean score for work engagement at baseline was average (according to scores defined by Schaufeli & Bakker [7]). Therefore, aiming at an increase in work engagement is considered feasible, as well as relevant. An increase in vigorous physical activity in leisure time, and a decrease in sedentary behaviour at work, are also feasible and relevant. Especially relevant would be to decrease the number of people not engaging in vigorous physical activity at all. The mean number of minutes per week engaging in vigorous physical activity and its non-normal distribution is comparable to a certain degree to findings in previous research [43] (mean = 70, sd = 168). Workers involved in the needs assessment indicated that they thought body weight was less important than mental health and well-being. The percentage of overweight and obese workers in the study population, 29% and 9%, respectively, was consistent with the Dutch working population, which is 31% overweight and 6% obese [58]. This is remarkable, however, since according to the same study, the prevalence of overweight and obesity among Dutch workers with scientific professions is significantly lower than the Dutch overall prevalence, namely 25.7% and 4.2%, respectively. Not all workers at the research institutes and included in this study perform scientific work, which may explain this discrepancy.

When making the choice for mindfulness training as a strategy of self-regulation, the need for cognition was assumed to be high. This was indeed the case, as the study population's need for cognition was relatively high (3.67-3.75 on a scale from 1 to 5).

Because the baseline characteristics were congruent with the findings in the needs assessment and the expectations on which the intervention was developed, we concluded that the systematic development based on the IM protocol produced a potentially appropriate intervention program, meeting the needs of this target population.

Furthermore, the baseline characteristics show that the intervention and control group did not differ significantly. Therefore, we concluded that randomization was successful, although for vigorous physical activity the groups did differ at baseline and this should be kept in mind when evaluating the effects.

### Strengths and Limitations

The first strength of the Mindful VIP study is targeting two threats to worker's health in the Western society: mental health problems and overweight as a result of a disturbed energy balance. A second strength of this study is that by developing the intervention in a systematic way, based on the IM protocol, the intervention is theory-based and the target population was involved in the development as well. As a result, the intervention is likely to be adequate to meet the needs of the target population, hopefully resulting in good compliance. Discussing the constraints of the implementation of the program and compliance with the target population decreases the likelihood of barriers in adoption of the program. Furthermore, the effectiveness will be evaluated in a two-armed RCT with two follow up measurements up to one year. Another strength of the study is that anthropometric measurements are conducted objectively, and physical activity and sedentary behaviour data are collected using a combination of subjective and objective methods.

This study also has limitations. As the intervention was developed for and by the target population, it is possible that this intervention is only suitable for the present study population. This means that the external validity might be limited. However, we feel that the intervention and study's results might be applicable to many other highly educated office workers as well. The two research institutes are comparable to the extent that they both conduct policy-based research, and mainly include office workers who perform mentally demanding work.

Another limitation of the study design is that blinding participants is not possible. On the one hand, the likelihood of a spill-over effect (i.e. contamination) is expected to be negligible, because the training and e-coaching only is accessible for those in the intervention group. On the other hand, all workers, for example, might take fruit when informed by colleagues from the intervention group. When evaluating the effects, this should be considered.

Further, the intervention only aims at the individual worker. Ideally, a combination of individual and environmental changes was included. A meta-analysis of the effects of worksite physical activity and dietary interventions have namely shown greater effects on weight related outcomes for combined interventions compared to interventions based on one component (i.e. either individual or environmental changes) [59]. Nevertheless, because of the randomisation at individual level, possibilities to achieve and evaluate environmental changes within the Mindful VIP study were limited. Within these limitations, however, some environmental changes have been made on a social level, generating support in the group training, and in the physical environment by providing fruit. Concerning work engagement, on the other hand, it has been hypothesized that changes in environmental resources only yield short term effects because workers tend to quickly accommodate changes, which they have not initiated themselves based on their personal resources [34]. Therefore, it would be more beneficial to aim principally at the personal resources of work engagement and the individually based intervention targeting work engagement in the present study thus meets that hypothesis. However, this raises the question of whether the responsibility to be engaged or feel well at work can entirely be abdicated on the worker, especially since it is work-related--thus there is a need for further research.

## Conclusion

The Mindful VIP program was developed to improve the work engagement and EBRB of workers performing mentally demanding, sedentary work. The results of the needs assessment during the systematic development of the intervention were consistent with the findings at baseline measurement. It is therefore likely that this program fits the target population well, increasing the likelihood of compliance and effectiveness. To determine the (cost-) effectiveness of this program, a RCT will be conducted. Related outcomes such as mental health, vitality, BMI, general health perception, need for recovery and work-related outcomes will also be examined. Results of the RCT will be available in 2012.

## Competing interests

The authors declare that they have no competing interests.

## Authors' contributions

All authors contributed to the conceptual design of the study and intellectual input into the design of this paper. JvB performed data collection, analysed data and drafted the manuscript. KIP, CRLB, PMB and AJvdB provided support during the development of the Mindful VIP program. All authors contributed to the further writing of the manuscript and read and approved the final version of the manuscript.

## Pre-publication history

The pre-publication history for this paper can be accessed here:

http://www.biomedcentral.com/1471-2458/11/736/prepub
